# An Intensive Approach to Improving Diversity, Equity, and Inclusion in an Academic Emergency Department

**DOI:** 10.5811/westjem.2022.3.55007

**Published:** 2022-06-06

**Authors:** Pamela Young, Dorcas Boahema Pinto, Shellie Asher, Sara DeSanctis, Karen Gardner, Sean Geary, Heather Long, Jennifer Pelesz, David Peltier, Lorraine Thibodeau, Denis Pauze

**Affiliations:** Albany Med Health System, Department of Emergency Medicine, Albany, New York

## Abstract

A healthcare workforce that demonstrates cultural competence and humility while reflecting the diversity of the surrounding community has the potential to significantly benefit the patient population it serves. In this context and given numerous societal influences and the events of 2020, the leadership of the Department of Emergency Medicine at Albany Medical Center recognized the need to promote diversity, equity, and inclusion (DEI) in multiple areas. These included premedical education, medical education, postgraduate medical education, faculty development, staff satisfaction, and patient care. The department formed a DEI taskforce that developed an ongoing, multipronged, interdisciplinary approach to address these important aspects of our work and clinical environment with the goals of improving staff wellbeing, reducing burnout, and promoting the health of our community. Our experience is shared here to illustrate how a small, dedicated team can implement a variety of DEI initiatives quickly and with relatively little cost at a large academic medical center.

## INTRODUCTION

A healthcare workforce that demonstrates cultural competence and humility while reflecting the diversity of the surrounding community has long been demonstrated to have significant potential to benefit the patient population it serves.^1^,^2^ Albany Medical Center (AMC) is a private, not-for-profit medical college and academic medical center that has provided medical education and patient care since the 1840s in a city 150 miles north of Manhattan in New York State. The AMC Level I Adult Trauma and Emergency Care Center and Level II Pediatric Emergency Department (ED) see approximately 80,000 patients per year and serve a very large catchment area, incorporating 25 counties in a 150-mile radius with 2.9 million persons in urban, suburban, rural, and austere environments. Because of this large catchment area, AMC is the busiest trauma center in the state.^3^

**Figure 1:**
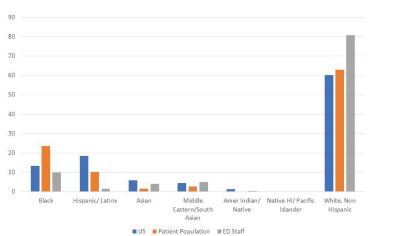
Comparison of population to staff representation by percentage. *HI*, Hawaiian; *ED*, emergency department.

**Figure 2:**
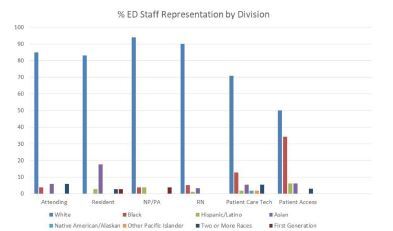
Ethnic/racial make-up of emergency department staff by division at Albany Medical Center. *ED*, emergency department; *NP*, nurse practitioner; *PA*, physician assistant; *RN*, registered nurse.

The patient population is a diverse mix of White (60%), Black or African American (23%), Asian (5%), and Hispanic (5%) populations including a significant proportion (13.2% in 2018) of foreign-born immigrants and refugees from countries in Central America, Africa and Asia, among others. Median household income in the metropolitan census area is $45,500, and 10–20% live below the poverty line (2019 data).^4^,^5^ Department staff include 45 faculty, 36 residents, 24 nurse practitioners and physician assistants (NP/PA), 125 nurses, 55 technicians, and 48 clerical staff in addition to a variable number of scribes, respiratory therapists, social workers, housekeeping staff, and rotating students. While non-clinical ED staff approximately reflect the surrounding community demographics, the clinical staff are largely White, with only two of the NP/PA group and two faculty identifying as from groups historically underrepresented in medicine (URiM). Staff representation compared to the surrounding population and by role are illustrated in Figures 1 and 2.

The summer of 2020 witnessed an historic shift in how citizens of the United States saw themselves and their neighbors, unfortunately sometimes resulting in animosity and violence. Protests and social justice gatherings roiled the country amid a worldwide pandemic. Throughout the year, AMC saw significant changes in its patient population with increasing volumes of coronavirus disease 2019 (COVID-19) patients in the spring, including dozens transferred to Albany to help decompress an overwhelmed New York City hospital system. Local and regional events in the summer brought an increase in victims of community violence, while overall patient volumes were down as patients stayed home through medical emergencies to try to keep themselves safe.

In this national setting, emotions and tensions within the department were elevated. As a result of conversations between one of the Black medical staff in the department and the chair of the AMC Department of Emergency Medicine (EM), various like-minded staff members gathered to address the topics of diversity, equity, and inclusion (DEI).

## MISSION

As a first step, the group identified its mission: to promote an inclusive and equitable environment for all members of the workforce within our emergency department (ED), regardless of gender, race or ethnicity, sexual orientation, title, or position. Furthermore, we strive to provide an environment where our patients feel safe, respected, and understood, regardless of demographics or socioeconomic status; to acknowledge and celebrate patient and staff diversity; to identify and address bias that may exist, both conscious and implicit; and to encourage self-reflection by all members of our workforce.

Stakeholders in this endeavor are led by the department chair, who is an active member of the taskforce and is also involved on a national level as a member of the Association of Academic Chairs of Emergency Medicine Diversity, Equity, and Inclusion Workgroup. The Chief Officer of Diversity and Inclusion for AMC has also been involved, providing input about the overall vision for DEI in the institution and facilitating conversation and collaboration. Active taskforce members include eight faculty physicians, three NP/PAs, two resident physicians, and two patient access representatives (non-clinical support staff). Five members identify as historically URiM. Ad hoc participants in outreach events have included registered nurses, additional patient access staff, medical technicians, respiratory therapists, and emergency medical services personnel. Our most important stakeholders are our patients and the Albany community at large.

## GOALS AND OBJECTIVES

**Figure 3:**
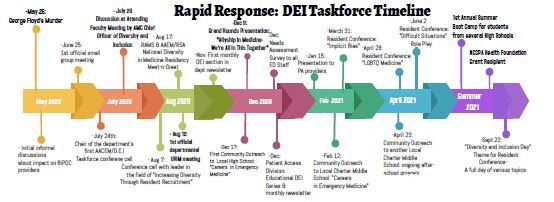
Overview of events sponsored by Albany Medical Center’s Diversity, Equity and Inclusion Taskforce (May 2020–September 2022). *DEI*, diversity, equity, and inclusion; *RAMS*, Society for Academic Emergency Medicine Residents and Students; *AAEM/RSA*, American Academy of Emergency Medicine Resident and Student Association; *PA*, physician assistant; *LGBTQ*, lesbian, gay, bisexual, transgender, and queer; *NCCPA*, National Commission on Certification of Physician Assistants; *BIPoC*, Black, indigenous and people of color; *AACEM*, Association of Academic Chairs of Emergency Medicine.

In support of this mission, several priorities for our taskforce were established: needs assessment; cultivating an environment of inclusion and representation; promoting individual and collective growth in DEI-related attitudes and skills; recruitment and retention of a diverse workforce; outreach to local middle schools and high schools to establish long-term relationships and encourage interest in health-related professions; and developing relationships with our community to build trust in our healthcare system. These priorities are furthered by the following initiatives (also depicted in Figure 3).

Goal: To examine the current work environment and identify the concerns of department personnel to prioritize areas of focus for improvement efforts.

### Initiative 1: Needs Assessment Among Emergency Department Staff

Preliminary informal conversations suggested that staff with a higher proportion of Black, indigenous, and people of color (BIPoC) representation had a high rate of experiencing and witnessing bias, microaggressions, or harassment directed toward themselves or toward patients of certain races or socioeconomic classes, creating tension and an uncomfortable work and patient-care environment. A survey was sent to all staff to further explore these issues, develop a baseline understanding of the ED environment and determine future areas of focus. The survey was distributed to all clinical and non-clinical staff whose jobs are primarily based in the ED.

Preliminary review of responses identified several areas of improvement. Overall, most of the respondents felt that their suggestions for improvement are heard and that the department genuinely desires to create a safe and equitable environment. The overwhelming majority agreed with the promotion of DEI as an appropriate goal for the department. Areas of opportunity identified included encouraging interprofessional communica-tion and cultivating an environment of respect toward all staff and patients. These results are being used to help guide efforts for intervention and will be reassessed to monitor progress. The survey also indicated that most staff are interested in learning to recognize bias, address these issues, and improve the work and patient-care environment.

### Initiative 2: Use of Resident Survey Data in Program Development

Starting in the 2019–2020 academic year the Accreditation Council for Graduate Medical Education increased its focus on issues of diversity, health equity, and inclusion for residency programs, now including questions relating to these topics on annual trainee and faculty surveys. This initial survey data will provide a baseline and opportunity for ongoing assessment of progress in resident and faculty perception of preparation for interaction with diverse individuals, inclusiveness of the work environment, and program efforts to recruit and retain a diverse workforce as we implement our DEI initiatives. Additionally, an institutional needs assessment headed by the Chief Officer of Health Equity, Diversity and Inclusion was carried out in the summer of 2020, with medical students surveying residents across departments about allyship and previous training received on this topic. The responses revealed a lack of formal training around allyship and a perception of this topic as a significant gap in education.

Goal: To cultivate an environment of inclusion and representation within our department for patients, visitors, and staff.

### Initiative 3: Staff Newsletter Diversity Highlights

As part of the goal to promote a culture of inclusivity and maintain healthy relationships between clinicians, the department chair launched a monthly newsletter that highlights achievements of staff members and provides brief educational articles. A section of this newsletter has been dedicated to the DEI taskforce and includes education, links to webinars and presentations, and a calendar that highlights upcoming presentations. In addition, our Patient Access group has started including DEI-related articles in their divisional weekly newsletter.

Goal: To promote individual and collective growth in DEI-related attitudes and skills among staff through education on cultural diversity, discrimination, implicit bias, and social determinants of health.

### Initiative 4: Resident and Staff Didactic Curriculum Development

Several new sessions are being incorporated into weekly didactics for the EM residency, with all members of the ED environment, including students and other clinical and non-clinical staff, also invited to participate. Four sessions were developed for the 2020–2021 academic year.

### Session 1: “Allyship in Medicine – We Are ALL in This Together”

This first session in the educational series was presented in December 2020 and served as an introductory session to ED staff and students on the DEI taskforce, its mission, basic DEI terminology, and the concept of allyship, which was identified as a curricular need based on previously described needs-assessment data. This one-hour, case-based presentation was developed by a group of medical students and modified for the ED setting by members of the DEI taskforce. It was presented by an interdisciplinary group including students, staff, and the Chief Officer for Diversity and Inclusion. A pre- and post-session survey demonstrated improved understanding of allyship and how to respond to episodes of bias or discrimination witnessed in the clinical and educational environment.

### Session 2: Implicit Bias

This one-hour presentation in March 2021 focused on the impact of implicit bias in the clinical and educational environment. It had been previously presented in other departments with positive feedback and generated vigorous discussion on the presence and potential impact of bias.

### Session 3: LGBTQ+ Healthcare

This one-hour presentation in April 2021 was based on best current evidence recommendations for healthcare and approach to the lesbian, gay, bisexual, transgender, queer + population, presented by a physician assistant with significant EM and public health experience.

### Session 4: Open Forum

In June 2021, this one-hour session consisted of role-play scenarios and provided time for open forum discussion to help solidify some of the information covered in the prior sessions.

### Initiative 5: Journal Club

In addition to these didactic sessions, EM journal clubs discussing key articles in DEI-related topics were planned throughout the academic year. Journal club is hosted by the residency program, and all clinical staff are invited.

Goal: To recruit and retain a diverse workforce to fully reflect the community and to improve patient care, education, and the work environment.

Albany Medical College has implemented a deliberate and targeted focus on holistic review of applicants with an emphasis on increasing successful applications for underrepresented minorities, first-generation students and other individuals facing socioeconomic barriers to progress in healthcare education and careers. As a result, the school has seen a significant increase in its URiM matriculants. The Graduate Medical Education Council is assessing institution-wide efforts to increase diversity and inclusion at the postgraduate training level as well. In conjunction with these institutional initiatives, the EM DEI Taskforce has embarked on related efforts in recruitment and retention across all levels of faculty and staff.

### Initiative 6: Faculty Recruitment, Retention, and Advancement

As previously noted, current ED staff demographics, particularly clinical staff, do not closely reflect the surrounding community and patient population. Recruitment and retention of healthcare workers outside major metropolitan areas poses unique challenges, and the specific factors impacting URiM faculty are unclear. With the understanding that those who have ties to the area are more likely to stay and invest in the community, we have developed short-, mid- and long-range goals toward developing pipeline relationships with community stakeholders.

We began with a critical assessment of our current faculty and having a frank, transparent discussion on factors that may contribute to lack of diversity. We acknowledge that candidates from all backgrounds need to see themselves and their potential contributions as valued, a “good fit” for the department and community, and with significant potential for growth and advancement. Our short-range goal is to increase awareness among stakeholders, explore barriers to recruitment and retention of traditionally URiM faculty, and take action to reduce those barriers. One issue identified was a lack of mentoring for junior faculty, and a formal mentoring program has been launched to promote faculty development and address impediments such as imposter syndrome.^6^

Mid- and long-range goals are to increase URiM representation in our faculty pipeline including the residency program, scribes, ED techs, and research associates. We hope to make progress in these pipelines through seed programs in local elementary through high schools and reaching out to premed advisors at local colleges to advertise these positions. In addition, the department chair and the chair of our departmental Promotions and Tenure Committee work closely with faculty to identify professional development needs and potential barriers to promotion in order to provide resources to retain faculty and promote success.

### Initiative 7: Nurse Practitioner and Physician Assistant Recruiting

As of 2019, less than 10% of PAs practicing in EM identify as URiM.^7^ Of our group of 20 full-time and six per diem NP/PAs, 85% are female, one is Black, and one is of Mexican descent. Three of this group are part of the DEI taskforce, and all are encouraged to participate in the various DEI education forums. The director of NP/PA operations, who is Black, has attempted to recruit a more diverse staff by advertising on social media platforms that are directed toward BIPoC, as well as reaching out to PA programs at historically Black colleges and universities (HBCU).

The department has sponsored a Physician Assistant Postgraduate Fellowship since 2010, an intense 12-month long program during which fellows see patients under faculty supervision in the ED and rotate through other services to learn EM-related skills. Approximately 50% of the program graduates have stayed on staff in the department in a full- or part-time capacity. The fellowship co-directors became involved with the DEI taskforce at its inception, with the goal to increase diversity in the program and to contribute to the depart-ment’s stated mission of promoting an environment of diversity, equity, and inclusion—for all. Targeted recruiting materials were sent to HBCU PA programs to garner the attention of URiM students to encourage them to consider the field of EM and our program in particular. Openings have also been advertised on various social media BIPoC-centered forums. Efforts to recruit a diverse applicant pool are ongoing, as well as assessment of the effects of these efforts. We are encouraged that for the current academic year, two-thirds of the program participants identify as URiM.

### Initiative 8: Resident Recruiting

The residency program director, assistant program directors, and chief residents have participated in DEI taskforce meetings and engaged in open dialogue concerning the current process of resident recruitment and potential ways to help match a more diverse group of students, including but not limited to the areas of race, gender identification and sexual orientation, and first-generation students. Residency leadership invited the input of an outside guest from a residency program that has successfully increased its representation of URiM residents.^8^ The residency recruitment process was examined to identify challenges in URiM applications and match rate. Candidate assessment now emphasizes a holistic approach that values overcoming adversity, community involvement/activism, and first-generation status.^6^,^8^

As part of residency recruiting, taskforce members were involved at all stages, including taking part in interviews, reaching out to URiM applicants after their interviews, participating in virtual “second look” events, and giving input to the candidate ranking process. For the 12 residents matched in 2021, two identify as URiM (previous years ~1/year on average) and three are first-generation college graduates (not previously tracked).

Goal: To increase exposure to careers in medicine for school-age children in underrepresented populations and to establish long-term relationships for pipeline programs.

### Initiative 9: “Career Day” in Emergency Medicine

Emergency medicine practice makes evident the importance of increasing diversity in our workforce to promote the health of the diverse population that we serve. Exposure to medicine during early school years can make a significant impact as to whether a student pursues this route later in life.^9^ Therefore, a program was initiated to reach out to students at local schools with a high matriculation rate of minority students. Our goal is to provide insight into the many future job opportunities in the ED and healthcare in general.

Members of the DEI taskforce approached representatives from nearby schools to offer sessions intro-ducing students to careers in the ED, with overwhelmingly positive response. Three sessions were held in the 2020–2021 academic year with local high school and middle school students via online platform. Several potential career opportunities were discussed including nursing, physician, NP/PA, respiratory therapy, medical technician, patient access staff, radiology technician, and emergency medical technician. Pathways to these careers were discussed and resources were provided for students interested in further information. Interactive presentation techniques were used, with students asking and answering questions using a polling system. Next steps include incorporating feedback and giving similar presentations at additional schools, and when it is appropriate per public health guidelines we will transition to a combination of in-person workshops and online events.

### Initiative 10: High School Student Medical “Boot Camp”

A “boot camp” experience was held in the summer of 2021, comprising a week of in-person programming to introduce high school students to careers in healthcare and get exposure to patient care principles. Students participated in hands-on learning and interactive workshops as well as sessions on career development, building a résumé, and interviewing skills. Students met physicians and staff across a variety of healthcare careers. Our goal through this program was to provide a lasting positive impression of healthcare for the students involved that will lead them to consider a career in medicine. We solicited feedback from participants and received a grant from the National Commission on Certification of Physician Assistants Health Foundation^10^ to provide future programming (Figure 3).

Goal: To improve our overall interactions with our diverse patient population, increasing their trust in our department and medical system.

### Initiative 11: Sickle Cell Walk/Fundraiser

Sickle cell disease is a condition predominately affecting the BIPoC population with significant individual and community impacts. Despite these significant impacts, sickle cell disease does not get the level of attention or support garnered by some other less common diseases.^11^ We think it is important to support research and funding for a disease that significantly impacts our patient population and raise awareness among our staff. The department was a sponsor for an inaugural Sickle Cell Walk fundraiser in 2020, hosted by the Sickle Cell Clinic at the Melodies Center for Childhood Cancer and Blood Disorders at our institution, along with the Underrepresented Student Alliance at Albany Medical College. Our staff raised over $1000 to serve as a sponsor again for the event in 2021 and anticipate that this will be a continuing community partnership moving forward.

### Initiative 12: Interactions with Local Educators

A number of local schools serve predominately lower income, BIPoC students, with the largest high school having a racially, ethnically, and culturally diverse population. In addition to direct student interactions described above, educators and staff at these schools have been enthusiastic about interacting with our department, and two taskforce members have been invited to participate in the Business Panel at the urban public high school.

## LIMITATIONS

As a preliminary description of a multipronged approach to improvement in DEI in our department, specific outcome measurements are limited at this time. Proposed outcomes include recruitment/retention of faculty, trainees, and staff; retention of current staff; improvement in wellbeing measures; and others to be determined. Our hope is to assess the patient- and community-centered outcomes of these efforts in future papers.

## CONCLUSION

It is our hope that through this interdisciplinary, multipronged approach, we will see significant improvements in diversity, equity, and inclusion in our work, clinical, and educational environment. We share these efforts to demonstrate how a small group of motivated individuals can come together as a team to make a potentially large impact for our community at relatively small cost and look forward to continuing to assess and refine this process based on feedback and outcomes.
